# Image-Guided Monitoring of Mitochondria and Blood–Brain Barrier Dysfunction in Amyotrophic Lateral Sclerosis Mice

**DOI:** 10.34133/bmr.0162

**Published:** 2025-03-17

**Authors:** Do Won Hwang, Jinhui Ser, Konstantyn Ziabrev, G. Kate Park, Min Joo Jo, Shinya Yokomizo, Kai Bao, Atsushi Yamashita, Hoonsung Cho, Maged Henary, Satoshi Kashiwagi, Hak Soo Choi

**Affiliations:** ^1^ Gordon Center for Medical Imaging, Department of Radiology, Massachusetts General Hospital and Harvard Medical School, Boston, MA 02114, USA.; ^2^ Research and Development Center, THERABEST Co. Ltd., Seoul 06656, South Korea.; ^3^ Department of Materials Science and Engineering, Chonnam National University, Gwangju 61186, South Korea.; ^4^ Department of Chemistry, Center of Diagnostics and Therapeutics, Georgia State University, Atlanta, GA 30303, USA.

## Abstract

Early detection of amyotrophic lateral sclerosis (ALS) progression is critical for improving disease management and therapeutic outcomes. However, the clinical heterogeneity and variability in ALS symptoms often lead to delayed diagnosis and suboptimal therapeutic interventions. Since mitochondrial dysfunction is a hallmark of ALS, we hypothesized that monitoring mitochondrial function could serve as a reliable strategy for early diagnosis and therapeutic monitoring of ALS. To address this, we synthesized and characterized 2 novel near-infrared fluorophores, ALS04 and ALS05, designed to target mitochondria and lysosomes. Their physicochemical properties, serum protein binding, fluorescence characteristics, photostability, and pharmacokinetics were systematically evaluated. We found that benzothiazole-based fluorophores exhibit excellent mitochondrial targeting, optimal optical properties, biocompatibility, and favorable biodistribution in vivo. Interestingly, ALS04 showed superior mitochondrial accumulation compared to ALS05, despite their similar physicochemical properties. This enhanced accumulation can be attributed to the lower molecular weight and higher lipophilicity of ALS04. Real-time fluorescence imaging revealed a substantial reduction in ALS04 signals in mitochondrial-rich tissues such as brown fat, highlighting its potential for monitoring mitochondrial dysfunction in early-stage ALS. Furthermore, the detection of ALS04 in the mouse brain suggests its ability to monitor blood–brain barrier hyperpermeability, another key feature of ALS pathology. These findings establish ALS04 as a promising noninvasive imaging tool for monitoring biomarkers associated with ALS progression. Its ability to detect early-stage pathophysiological changes in an ALS mouse model highlights its potential for advancing our understanding of ALS mechanisms and facilitating the identification of novel therapeutic targets.

## Introduction

**Fig. 1.**
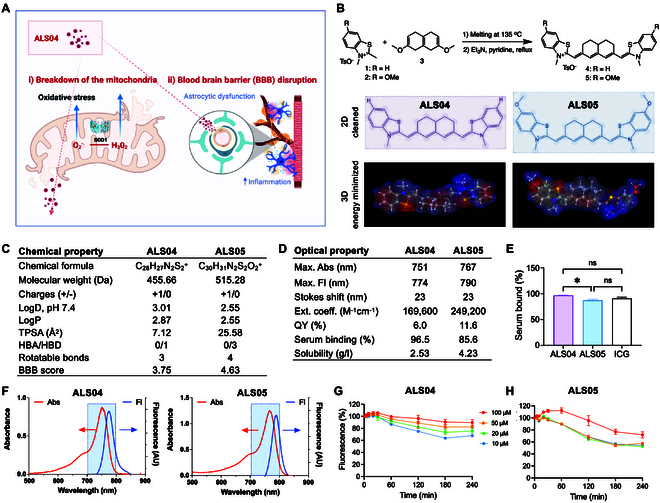
Synthetic scheme and optophysicochemical properties of ALS fluorophores. (A) Schematic representation of the overall study hypothesis. (B) Synthetic scheme for ALS fluorophores, including 2-dimensional (2D) and 3D chemical structures of ALS04 and ALS05. (C) Quantitative calculation of the physicochemical properties of ALS04 and ALS05, calculated using MarvinSketch 23.11 (ChemAxon). TPSA, topological polar surface area; HBA, hydrogen bond acceptors; HBD, hydrogen bond donors; BBB, blood–brain barrier. (D and E) Optical properties and results of serum binding and solubility for ALS04 and ALS05. Optical properties were determined in DMSO. Ext. coeff., extinction coefficient; QY, quantum yield; Abs, absorbance; Fl, fluorescence. Statistical analysis was performed using one-way ANOVA followed by Sidak’s test (*n* = 3, mean ± SD). (F) Representative absorbance and fluorescence spectra of ALS04 and ALS05. (G and H) Photostability pattern of ALS04 and ALS05 at varying concentrations in 5% BSA in saline, calculated by plotting the percentage of retained fluorescence (*n* = 3, mean ± SD).

The neurodegenerative disease known as amyotrophic lateral sclerosis (ALS) steadily progresses over time and is a fatal disease. It is characterized by the selective loss of upper and lower motor neurons, which causes voluntary muscle atrophy and parlay [1]. After a variety type of pathological changes at a molecular and cellular level, motor neurons begin rapidly degenerating at the late-onset stage, accompanied by symptomatic abnormality, including muscle wasting in ALS patients [2]. However, phenotypic variability makes proper diagnosis challenging and leads to therapy failure since disease progression is highly complex [3]. Therefore, biomarker-based diagnosis is crucial to determining the progression of the disease or the efficacy of a treatment intervention [4]. The known pathogenesis causing both familiar and sporadic ALS shares deposition of aggregated proteins [5], oxidative stress [6], hyper-excitotoxicity [7], metabolic disturbance [8], and mitochondrial dysfunction [9]. Mitochondria play a crucial role in cell survival and metabolism and their dysfunction and have been reported to be the primary early symptoms of severe neurodegenerative disorders, including ALS (Fig 1A) [10]. In addition, blood–brain barrier (BBB) breakdown caused by neurodegeneration is another common symptom of ALS and other neurodegenerative diseases (Fig. 1A) [11,12]. As an essential part of the central nervous system (CNS), the BBB tightly regulates the flow of nutrients and metabolites into the brain parenchyma via a transport system, preventing the entry of potentially hazardous substances. With the progression of the disease, the BBB becomes more permeable, allowing a more comprehensive range of molecules to pass through the CNS [13]. Therefore, mitochondrial dysfunction and BBB hyperpermeability could be sensitive biomarkers to monitor the progression of ALS. In particular, noninvasive imaging of these biomarkers would be a feasible strategy for timely monitoring of the disease progression.

Among current imaging modalities, near-infrared (NIR) fluorescence imaging emerged as a valuable tool for noninvasive and functional imaging of live tissues, as it can penetrate deeper into tissues than visible light, allowing for imaging of deeper structures [14,15]. With a proper choice of a contrast agent with specific targeting to the cell or organ and biocompatible physiochemical properties, NIR imaging features reduced nonspecific tissue uptake for the high target-to-background ratio (TBR) [16,17]. Such an agent requires the rational design of NIR fluorophore [18]. These rationally designed NIR fluorophores have been proven beneficial in broad applications such as image-guided surgery [19] and organ-targeted drug delivery [20]. However, there is a paucity of fluorophores to monitor mitochondrial function and BBB permeability and be suitable for the assessment of ALS progression in the clinic. In this study, we established an ALS-related pathology-targeting system using specific NIR fluorophores via real-time NIR fluorescence imaging of relevant biomarkers in the ALS mouse model. Since the biomarkers for ALS are shared with other neurodegenerative diseases, the rationally designed fluorophore has the potential to enable a precise evaluation of the disease progression and timely therapeutic intervention for a wide array of neurodegenerative diseases.

## Materials and Methods

### Synthesis of ALS-targeted heptamethine cyanine fluorophores

All chemicals and solvents were of American Chemical Society grade or high-performance liquid chromatography (HPLC) purity and were used as received. Dipyrrolidino(*N*-succinimidyloxy) carbenium hexafluorophosphate (HSPyU), *N,N*-diisopropylethylamine (DIEA), *N*-hydroxysuccimide (NHS), and ethyl acetate were purchased from Fisher Scientific (Pittsburgh, PA), Sigma-Aldrich (Saint Louis, MO), or Acros Organics (Morris Plains, NJ). Other solvents were purchased from VWR International (West Chester, PA) and American Bioanalytic (Natick, MA). The synthesis of heptamethine cyanines **4** (ALS04) and **5** (ALS05) was made based on the procedure published by Sakharov [21]. A mixture of corresponding individual benzothiazolium tosylate salts **1** and **2** (1 mmol) and 2,7-dimethoxy-1,4,5,8-tetrahydronaphthalene **3** (1.1 mmol) was melted at 135 °C for 15 min and then cooled to room temperature. The second equivalent of benzothiazolium salt (1 mmol) was added to the reaction mixture, followed by pyridine (10 ml) and triethylamine (0.5 ml), and the resulting mixture was refluxed for 1 h. After cooling the reaction mixture to room temperature, it was allowed to stand overnight. The crystalline of compounds **4** and **5** was filtered off and washed with pyridine (3 × 10 ml) and EtOH (3 × 5 ml) to obtain analytically pure compounds.

### Optical and physicochemical properties

MarvinSketch (ver. 23.11) and InstantJChem (ver. 23.17) calculator plug-ins (ChemAxon, Budapest, Hungary) were used to calculate in silico physicochemical property, such as molecular weight (MW), the partition and distribution coefficient (Log*P* and Log*D* at pH 7.4), surface molecular charge, hydrophobicity, the hydrogen bond acceptors/donors (HBA/HBD), the acid dissociation constant (p*K*_a_), the topological polar surface area (TPSA), and BBB score. The BBB score is determined by 5 key physicochemical descriptors, including the number of aromatic rings, the number of heavy atoms, MWHBN (a parameter derived from MW and the number of HBA/HBD), TPSA, and p*K*_a_ [22]. The final score is computed using stepwise methods for descriptors with discrete values and polynomial weight functions for descriptors with continuous ranges (ChemAxon). The absorbance and fluorescence emission spectra of ALS04 and ALS05 at varying concentrations were measured in dimethyl sulfoxide (DMSO) using HR2000 absorbance spectrometers (200 to 1,100 nm) and USB2000FL fluorescence spectrometers (350 to 1,000 nm) from Ocean Optics (Dunedin, FL). Fluorescence emission spectra were collected under NIR excitation using a 760-nm laser pointer (Opcom Inc., Xiamen, China). To determine the quantum yield (QY) of the fluorophores, indocyanine green (ICG) in DMSO (QY = 13%) was used as a reference [23]. The fluorescence emission spectrum was integrated and plotted as a function of absorbance at 755 nm, and a linear fit was applied to determine the slope between fluorescence intensity and absorbance. The NIR fluorescence QY was calculated using the following equation:

$${\mathrm{QY}}_{\text{sample}}={\mathrm{QY}}_{\text{ref}}\times \frac{{\text{slope}}_{\text{sample}}}{{\text{slope}}_{\text{ref}}}\times {\left(\frac{n_{\text{sample}}}{n_{\text{ref}}}\right)}^{2}$$ (1)

where *QY*_*sample*_ and *QY*_*ref*_ are the QY of ALS04 or ALS05 and ICG, respectively. *n*_*sample*_ indicates ALS04 or ALS05, *n*_*ref*_ means ICG, and all the refractive indexes are 1.4793 using the same DMSO solution. To assess the solubility of ALS04 and ALS05, we measured the extinction coefficients of each fluorophore in 5% bovine serum albumin (BSA) in saline as a biological solution. A small, accurately weighed amount of each fluorophore was added to a minimal volume of solvents, and the saturated solution was then vortexed and sonicated for 5 min to ensure complete dissolution of the solids. Following this, the solution was centrifuged at 1,500*g* for 10 min, and the absorbance of the supernatant was measured. The solubility (g/l) was calculated by multiplying the maximum absorbance-derived concentration by the MW of each fluorophore.

### Serum binding and photostability test

The serum protein binding assay was performed using a rapid equilibrium dialysis (RED) device purchased from Thermo Fisher Scientific (Waltham, MA). Samples were prepared in 10% fetal bovine serum (FBS) with fluorophores at a concentration of 20 μM and then were added into a sample chamber and the dialysis buffer [phosphate-buffered saline (PBS); pH: 7.4] into a buffer chamber. The chambers were then incubated at 37 °C on a shaker for 8 h. The concentration of the fluorophore in each chamber was determined by measuring the absorbance value on Cytation 5 (BioTek, Winooski, VT), and the percentage of the bound (% bound) and unbound compound for each sample was calculated.

To investigate the photostability of ALS04 and ALS05, 10 mM stock solutions were diluted to obtain working solutions, with concentrations of 100, 50, 20, and 10 μM in 5% BSA solution, which were then dispensed into a black 96-well plate. Subsequently, the 200-μl working solutions were exposed to continuous irradiation using a 760-nm laser diode at 4 mW/cm^2^, with white light (400 to 650 nm) at 40,000 lux, and imaged for 240 min. The fluorescence (%) was calculated by analyzing the measured values with ImageJ v1.52 (National Institutes of Health, Bethesda, MD, USA) using images captured at each time point. The data, presented as mean ± SD, assessed stability by measuring regions of interest (ROIs) and comparing fluorescence intensity against the initial fluorescence signal.

### Subcellular localization study

Human neuroblastoma SH-SY5Y cells [American Type Culture Collection (ATCC), Manassas, VA, USA] were cultured in a 1:1 mixture of Eagle’s minimum essential medium (EMEM; no. 30-2003, ATCC, Manassas, VA, USA) and F12K with 10% FBS and 1% penicillin-streptomycin solution. Cells were grown in a humidified atmosphere of 5% CO_2_ at 37 °C. The medium was replaced every 3 to 4 d, and cells were subcultured using trypsin–EDTA when 70% to 80% confluent. All media and supplements were purchased from Gibco (Gaithersburg, MD, USA). To optimize the imaging condition in this study, cells were incubated at 37 °C for 10 to 30 min in the presence of 0.1 to 1 μM of ALS04 and ALS05. After washing, the cells were imaged using BioTek Cytation 5. Fluorescence intensity was quantified using the threshold analysis method implemented in the ImageJ software. SH-SY5Y cells were seeded on a 6-well plate (Corning Costar, no. 3516) at a density of 12,000 cells/well and incubated at 37 °C for 24 h. Then, 1 μM of ALS04 and ALS05 with Hanks’ balanced salt solution (HBSS; Lonza, no. 10543F) was added to cells and incubated for 30 min at 37 °C. The cells were then washed and stained with NucBlue (2 drops/ml, Live ReadyProbes Reagent, Hoechst 33342, Invitrogen no. R37605), Lysotracker Yellow (5 μM, Invitrogen, no. L12491), and MitoTracker Deep Red (0.5 μM) (Invitrogen, no. M22426) to stain lysosome and mitochondria for 30 min as per the manufacturer’s instruction. The cells were then washed with HBSS and imaged using BioTek Cytation 5. Mander’s colocalization coefficients were then determined using the Costes method to choose threshold values on ImageJ [24].

### Cellular uptake and cytotoxicity assay

SH-SY5Y cells were seeded on a 24-well plate (Corning Costar, no. 3524) and incubated at 37 °C for 48 h. To determine the role of membrane transporter in ALS04 retention, SH-SY5Y cells were pretreated with 250 μM sulfobromophthalein disodium (BSP; MedChemExpress, HY-D0217) at 37 °C for 20 min. Cells were then incubated with 1 μM of ALS04 and NucBlue (Invitrogen, Hoechst 33342, no. R37605) for 20 min at 37 °C. To evaluate the contribution of diffusion across the plasma membrane, cells were also incubated at 4 °C for 30 min, followed by incubation with 1 μM ALS04 at 4 °C for 20 min. After washing, the cells were imaged using BioTek Cytation 5. The fluorescence intensity of each cell was determined using ImageJ.

Cell viability was assessed using Cell Counting Kit-8 (CCK-8) from Dojindo Molecular Technologies Inc. (Kumamoto, Japan). Briefly, SH-SY5Y and NIH3T3 cells were seeded into 96-well plates at a density of 2,500 cells per well. The cells were then treated with 0 to 20 μM of each fluorophore in growth medium for 24 h, and 10% CCK-8 solutions were added to each well. The plates were incubated at 37 °C for 4 h, and absorbance was measured at 450 nm using a SpectraMax M5 plate reader (Molecular Devices). Cell viability (%) = (*A*_*sample*_ − *A*_*b*_)/(*A*_*c*_ − *A*_*b*_) × 100, where *A*_*sample*_, *A*_*b*_, and *A*_*c*_ denote the absorbance reading of sample, blanks, and negative control wells, respectively.

### In vitro model of mitochondrial dysfunction

SH-SY5Y cells were seeded into 6-well plates (Corning Costar, no. 3516) at a density of 12,000 cells/well overnight. Cells were maintained at 37 °C in a humidified atmosphere of 5% CO_2_. To model mitochondrial dysfunction, undifferentiated and differentiated SH-SY5Y cells were incubated with 30, 125, and 500 μM 1-methyl-4-phenylpyridinium (MPP^+^; Neurotoxin agent, Cayman, no. 16958) in the medium for 24 h. The control groups for undifferentiated and differentiated SH-SY5Y cells were treated with the same medium without MPP^+^. The cells were then washed with 10% FBS/HBSS. After washing, cells were incubated with 1 μM of ALS04, MitoTracker Deep Red, and NucBlue for 30 min. The cells were imaged using BioTek Cytation 5. The fluorescent intensity of each cell was measured using ImageJ.

### Biodistribution and pharmacokinetic study

Six-week-old CD-1 mice were used to quantify the blood clearance rate and urinary excretion. Mice were injected with 25 nmol of ALS04 or ALS05 in saline containing 2.5% PEG400 (polyethylene glycol, molecular weight 400) and 5% BSA via intravenous injection under isoflurane anesthesia, and blood was collected in heparinized capillary tubes (Fisher Scientific, Pittsburgh, PA) by tail nick before injection and at 1, 3, 5, 10, 30, 60, 120, 180, and 240 min post-injection and then stored in an icebox to prevent clotting. The supernatants of collected blood were filled into capillary microtubes (Fisher Scientific) after centrifugation. Mice were euthanized with CO_2_ inhalation to image organs and collect urine from the bladder 4 h post-injection. Collected urine samples were also placed into capillary microtubes. The fluorescence intensities of the microtubes were measured by NIR imaging along with a set of standard ALS04 or ALS05 samples of known concentrations to analyze the pharmacokinetics. Pharmacokinetic analysis was performed using the 2-compartment model (or biexponential decay) to estimate distribution (*t*_1/2α_) and elimination (*t*_1/2β_) blood half-life, the volume of distribution, and clearance. Results were presented as a biexponential decay curve.

### Targeting study

For biodistribution and targeting studies, 12-week-old B6SJLF1/J (SOD1^G93A^)1Gur male mice (25 to 30 g) were purchased from the Charles River Laboratories (Wilmington, MA). Animals were housed in an AAALAC-certified facility [Massachusetts General Hospital (MGH) #D16-00361], and all animal studies were performed in accordance with the Public Health Service Policy on Humane Care of Laboratory Animals and approved by the MGH Institutional Animal Care and Use Committee (IACUC) (#N2016000136). To confirm that mice did not show any overt clinical symptoms, before NIR imaging, we conducted a simple hanging wire behavior test when the age of SOD1^G93A^ male mice was between 14 and 16 weeks [25]. Each mouse was set on the wire lid of a typical housing cage, which was then turned on its side. It was timed how long it took after the test started for the mouse to stand with at least 2 limbs on the lid. The longest latency was noted after the animals were given 3 chances to stand for a maximum of 3 min per trial. The hemizygous SOD1^G93A^ mice were selected via genotyping polymerase chain reaction (PCR) assay. Mice were fed chlorophyll-free mouse chow (VWR International. Radnor, PA) at least 5 d prior to imaging to minimize autofluorescence. To determine in vivo targeting and biodistribution of ALS04 and ALS05, 25 nmol of each fluorophore was prepared in saline with 5% (w/v) BSA and injected retro-orbitally under isoflurane anesthesia into the ALS mouse model.

### In vivo NIR imaging

For all real-time intraoperative imaging, a standardized imaging protocol was employed throughout the procedure using our custom-made imaging system, as previously described [26]. Briefly, imaging utilized a 760-nm excitation source at a fluence rate of 4 mW/cm^2^ combined with white light illumination (400 to 650 nm) at 40,000 lux. Camera exposure time was adjusted as needed to accurately capture the biodistribution of the fluorophores. Mice were maintained under general anesthesia using isoflurane or a combination of ketamine and xylazine (Webster Veterinary, Fort Devens, MA), and a midline incision was performed to expose the abdominal cavity. For imaging, a general field of view (FOV) of 5 cm in diameter was used to include the pancreas head, duodenum, liver, and kidneys, while a close-up FOV of 3.3 cm in diameter focused specifically on the pancreas head and duodenum. Following imaging, the mice were euthanized using CO₂ inhalation. Major organs, including the heart, lung, liver, pancreas, spleen, kidney, duodenum, intestine, and muscle, were excised and imaged ex vivo to evaluate fluorophore biodistribution and excretion pathways. The signal-to-background ratio (SBR) was calculated as follows:

$$SBR=\frac{I_{ROI}}{I_{\text{Auto}}}$$ (2)

where $I_{ROI}$ denotes the average intensity of an ROI and $I_{\text{Auto}}$ represents the intensity of the muscle.

### Statistical analysis

Results are presented as mean ± SD for all analyses. Statistical analyses were conducted using GraphPad Prism 9 (GraphPad, San Diego, CA) through 1-way or 2-way analysis of variance (ANOVA), followed by Sidak’s multiple comparisons test. Statistical significance was denoted according to GraphPad conventions: not significant (ns), *P* > 0.05, *P ≤ 0.05, **P ≤ 0.01, ***P ≤ 0.001, and ****P ≤ 0.0001. Fluorescence intensity (%) and colocalizing pixels (%) were quantified using ImageJ software based on the measured values.

## Results

To evaluate the pathophysiological changes of tissues and organs in the early stage of ALS, a series of NIR fluorophores, including ALS04 and ALS05, possessing a heptamethine core to emit fluorescence around 800 nm, were designed and synthesized. Figure 1B outlines the synthesis of heptamethine cyanines **4** (denoted as ALS04) and **5** (ALS05) with decalin in the polymethine backbone containing a hydrogen atom **1** or a methoxy atom **2**, respectively. The details of ^1^H- and ^13^C-NMR (nuclear magnetic resonance) chromatography and HPLC spectroscopy of ALS04 and ALS05 are shown in Figs. S1 and S2, respectively. Next, the physicochemical and optical properties of the ALS-targeted fluorophores were investigated. As shown in Fig. 1C, ALS04 and ALS05 show modest hydrophobicity (Log*D* at pH 7.4 = 3.01 and 2.55; Log*P* = 2.87 and 2.55, respectively). The BBB score of ALS04 and ALS05, calculated by MarvinSketch 23.11 (ChemAxon), was found to be 3.75 and 4.63, respectively. Further evaluation of protein–fluorophore interactions in the bloodstream was conducted using the rapid equilibrium dialysis (RED) technique (Fig. 1D and E). Both ALS04 and ALS05 demonstrated NIR fluorescence, with excitation and emission maxima at 751/767 nm and 774/790 nm, respectively. They also exhibited excellent optical properties for intraoperative imaging, with high molar absorptivity of 169,600 and 249,200 M^−1^ cm^−1^ and QY of 6.0 and 11.6%, respectively, comparable to ICG with a molar absorptivity of 232,000 M^−1^ cm^−1^ and QY of 13% in DMSO, respectively. Interestingly, ALS04 and ALS05 exhibited similar serum binding compared to the Food and Drug Administration (FDA)-approved reference standard ICG, despite having lower Log*D* values (ICG: Log*D* at pH 7.4 = 4.91; Log*P* = 6.05) [27]. Due to their strong binding affinity, ALS04 and ALS05 are expected to be slowly released as unbound forms in tissue. The solubility of ALS04 and ALS05 was found to be 2.53 and 4.23 g/l in 5% BSA solution as a biological buffer, respectively, indicating their suitability for use in aqueous environments relevant to biological applications (Fig. 1D). As shown in Fig. 1G and H, ALS04 and ALS05 exhibited over 90% and 70% photostability, respectively, in 5% BSA solution under NIR imaging conditions for up to 240 min. However, at a concentration of 10 μM, photostability decreased to 60% for ALS04 and 55% for ALS05. These findings suggest that ALS-targeted fluorophores possess optimal physicochemical and optical properties for noninvasive NIR imaging applications.

Prior to conducting the in vitro cellular assays, we evaluated the toxicity of ALS04 and ALS05. Human neuroblastoma SH-SY5Y cells and fibroblast NIH3T3 cells were incubated with the fluorophores, and cell viability was assessed. The IC_50_ for both fluorophores was approximately 1.3 μM (Fig. S3), indicating their safety for imaging applications. To investigate the targetability of ALS04 and ALS05, we performed in vitro live-cell imaging to analyze subcellular localization in cultured SH-SY5Y cells. Based on optimization studies (Fig. S4), cells were incubated with 1 μM of the fluorophores for 30 min prior to imaging. ALS04 was designed to target mitochondria by leveraging the mitochondrial membrane potential (∆Ψ_m_), a widely used strategy for mitochondrial targeting agents. The positive charge of ALS04, as a delocalized lipophilic cationic molecule, facilitated its preferential accumulation in mitochondria, enhancing targeting specificity [28].

**Fig. 2.**
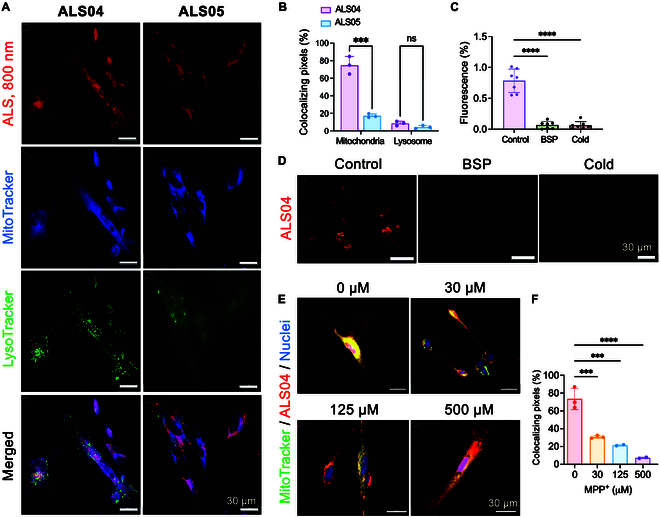
Subcellular localization and retention of ALS fluorophores. (A) SH-SY5Y cells were incubated with 1 μM of ALS04 or ALS05 for 30 min at 37 °C. Lysosomes and mitochondria were stained with LysoTracker and MitoTracker, respectively. Scale bar, 30 μm. (B) The percentage (%) of colocalizing pixels was calculated by dividing the overlap region of ALS fluorophores with LysoTracker or MitoTracker in merged images. Data were analyzed using 2-way ANOVA followed by Sidak’s tests (*n* = 3, mean ± SD). (C) Inhibition of ALS04 cellular uptake. SH-SY5Y cells were pretreated with BSP for 20 min before incubation with 1 μM ALS04 for 20 min. Alternatively, cells were incubated at 4 °C for 30 min prior to ALS04 exposure at 4 °C for 20 min. Statistical significance was determined using one-way ANOVA followed by Sidak’s tests (*P < 0.05, *n* = 3, mean ± SD). (D) SH-SY5Y cells were pretreated with inhibitor KN93 or oligomycin for 45 min, followed by incubation with 1 μM ALS04 in 10% FBS in HBSS for 30 min. Scale bars, 100 μm. (E) SH-SY5Y cells were preincubated with various concentrations of the neurotoxin 1-methyl-4-phenylpyridinium (MPP^+^) for 24 h and then stained with 1 μM ALS04 for 30 min. Scale bars, 30 μm. (F) Colocalizing pixels (%) were determined as the overlap area (yellow) divided by the total ALS fluorophore signal (red) and mitochondria signal (green). Data were analyzed using one-way ANOVA followed by Sidak’s tests (*n* = 3, mean ± SD).

Fluorescence signals were observed in both mitochondria and lysosomes (Fig. 2A and B), consistent with previous studies showing that cyanine derivatives and other lipophilic cations accumulate in these organelles [29,30]. While ALS04 and ALS05 exhibited similar colocalization with lysosomes at 8.5% and 4.4%, respectively, ALS04 demonstrated significantly higher colocalization with mitochondria at 71%, compared to 18% for ALS05 (Fig. 2B; ***P = 0.0005). Despite the similar physicochemical properties of ALS04 and ALS05, the superior mitochondrial accumulation of ALS04 can be attributed to its lower MW and higher lipophilicity. Additionally, the data suggest that ALS04 has a slower dissociation rate from mitochondrial targets compared to its off-target interactions, resulting in predominant accumulation within mitochondria and reduced interactions with other cellular regions [31,32]. Together, these findings indicate that ALS04 selectively accumulates and is retained in mitochondria and could be used to monitor mitochondrial function under NIR fluorescence imaging.

Based on these results, we further investigated whether ALS04 could monitor functional changes in mitochondria. Specifically, we hypothesized that the positively charged heptamethine fluorophore ALS04 is internalized by target cells via organic anion transporter peptides (OATPs) and accumulates in mitochondria based on mitochondrial function, making it a potential tool for monitoring ALS disease progression [33]. To prove this hypothesis, SH-SY5Y neuronal cells were pretreated with the OATP inhibitor BSP. BSP treatment resulted in a significant reduction in the fluorescence signal of ALS04 (Fig. 2C, ****P ≤ 0.0001), suggesting that OATPs are critical for ALS04 cellular uptake. Additionally, uptake of ALS04 was significantly reduced when cells were incubated at 4 °C, indicating that ALS04 relies on active transport rather than passive diffusion for cellular entry. As shown in Fig. 2E and F, treatment with MPP^+^, which is an inhibitor of mitochondrial complex I and has been widely used as a neurotoxin, led to a dose-dependent reduction in the colocalization of ALS04 with mitochondria. These results indicate that ALS04 preferentially accumulates in functional mitochondria. Overall, these findings demonstrate that ALS04 can detect mitochondrial dysfunction, further supporting its potential as a tool for monitoring mitochondrial activity and disease progression in ALS.

**Fig. 3.**
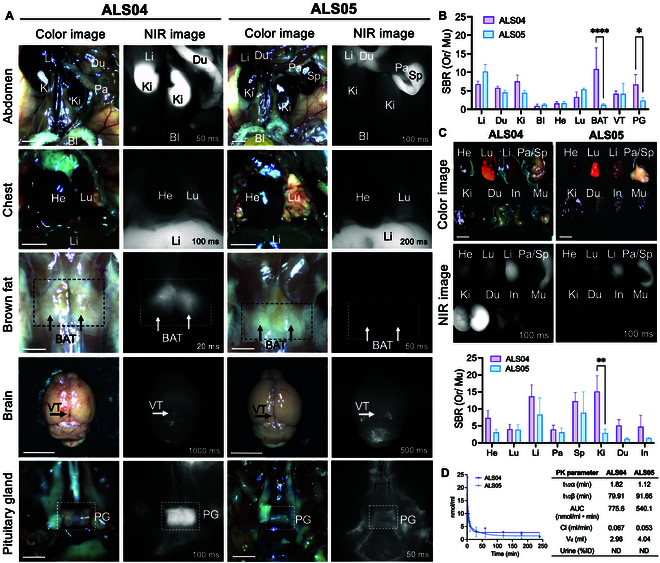
Biodistribution and clearance of ALS04 and ALS05 in CD-1 mice. (A) CD-1 mice were injected retro-orbitally with 25 nmol of ALS04 or ALS05 dissolved in 5% BSA in saline. NIR fluorescence imaging was performed, and organs were resected 4 h post-injection for further analysis. (B) SBRs of ALS04 and ALS05 in major organs were quantified from NIR fluorescence images using Sidak’s 2-way ANOVA (*n* = 3, mean ± SD). (C) SBRs of resected organs at 4 h post-injection were calculated by dividing the fluorescence signal intensity of major organs by that of the muscle. Li, liver; Du, duodenum; Ki, kidneys; BI, bladder; He, heart; Lu, lungs; BAT, brown adipose tissue; VT, ventral temporal region; PG, pituitary gland; Pa, pancreas; Sp, spleen; In, intestine; Mu, muscle. Scale bars, 5 mm. (D) Pharmacokinetics of ALS04 and ALS05 were evaluated following intravenous injection of 25 nmol of each fluorophore in 2.5% PEG400 and 5% BSA in saline into CD-1 mice.

Following these in vitro testing results, we hypothesized that ALS04 could monitor functional changes in mitochondria and the integrity of BBB in vivo (Fig. 1A), while ALS05 could serve as a negative control. To determine the in vivo biodistribution of the ALS-targeted fluorophores, the fluorophore was intravenously injected into mice, followed by intravital NIR imaging and ex vivo tissue biodistribution 4 h after injection. ALS04 showed relatively high SBR in the duodenum or intestine in addition to the kidneys, indicating both renal and hepatic clearance, while ALS05 displayed predominantly hepatic clearance (Fig. 3A). Little signal was found in the bladder for ALS04 and ALS05, indicating a relatively small contribution to the renal clearance route. Interestingly, ALS04 also showed highly selective accumulated signals in multiple tissues in brown adipose tissue (BAT) below the scapula and pituitary gland (PG) as compared to ALS05 (Fig. 3A and B; ****P ≤ 0.0001 for BAT and *P = 0.0324 for PG, respectively). On a note, mitochondria-enriched BAT requires an increase in mitochondrial energy expenditure for thermogenesis. These results show that ALS04 could accumulate in mitochondria in vivo after intravenous injection and could be used to assess mitochondrial function in tissues. Consistent with intravital NIR imaging, ex vivo tissue biodistribution results show a high signal of ALS04 in the gastrointestinal tract at 4 h post-injection, indicating rapid excretion from the liver and bile ducts (Fig. 3C). ALS04 showed a noticeably higher signal in the kidney compared to ALS05 (Fig. 3C). Next, the pharmacokinetic parameters of ALS04 and ALS05 were evaluated after a single intravenous injection. The blood concentration decay indicated a 2-compartment model with a blood half-life of 79.91 and 91.66 min and an area under the curve (AUC) of 775.6 and 540.1 nmol/ml·min, respectively, indicating that ALS04 and ALS05 display excretion through bile (Fig. 3D). The relatively short blood half-life of ALS04 allows for excreting unbound molecules promptly from the body, decreasing circulation time, nonspecific uptake, and potential toxicity. Based on the ability of ALS04 to image the peripheral tissue via mitochondria targeting, we performed a real-time NIR imaging of a mouse model of ALS. We adopted an ALS animal model that has a decreased copy number of the variant SOD1 transgene. Generally, the abnormal behavior of SOD1^G93A^ mice was initiated after 120 d [34].

**Fig. 4.**
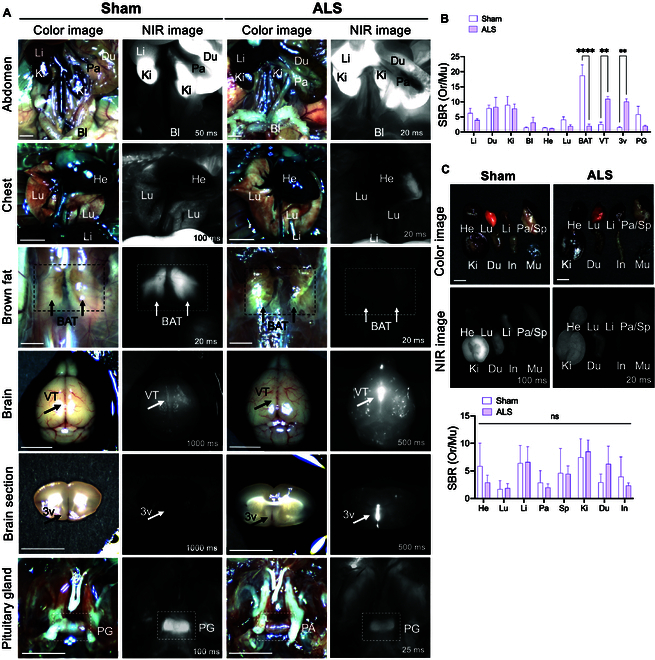
Biodistribution and targeting of ALS04 in ALS mice. ALS04 (25 nmol) in 10% BSA was injected retro-orbitally into sham and SOD1^G93A^ mice 4 h prior to imaging and resection. (A) Intraoperative color-NIR imaging of ALS04. (B) Quantitative analysis of the SBR in major organs relative to muscle was conducted using NIR imaging at 4 h post-injection, and statistical significance was determined using Sidak’s 2-way ANOVA (*n* = 3, mean ± SD). **P ≤ 0.01; ****P ≤ 0.0001. (C) Ex vivo color and NIR imaging were used to evaluate the biodistribution in major organs (*n* = 3, mean ± SD). ns, not significant; 3v, third ventricle. Scale bar, 5 mm.

To evaluate the potential of ALS04 to detect presymptomatic changes in ALS, the fluorophore was administered to 3-month-old asymptomatic SOD1^G93A^ mice, a well-established ALS model. Age-matched sham mice were used as normal controls. ALS04 uptake was significantly reduced in BAT of SOD1^G93A^ mice compared to sham mice at 4 h post-injection (Fig. 4A and B; ****P ≤ 0.0001). This reduction is attributed to reactive oxygen species (ROS)-mediated mitochondrial dysfunction in ALS mice, which impairs adenosine 5′-triphosphate (ATP) synthesis through defects in the electron transport chain and down-regulates mitochondrial functional genes (UCP1) in BAT. The functional abnormalities in BAT, which contribute to heat generation, result in compensatory skeletal muscle shivering in ALS mice [13]. As ALS04 selectively accumulates in functional mitochondria, the decreased fluorescence intensity in BAT indicates mitochondrial impairment in SOD1^G93A^ mice.

Additionally, BBB disruption is a hallmark of many neurodegenerative diseases that occur during early disease stages [11]. Hyperpermeable BBB has been reported in presymptomatic SOD1^G93A^ mice [12]. Consistently, significantly higher ALS04 signals were observed in the ventral temporal (VT) lobe and third ventricle (3v) of ALS mice compared to sham mice with intact BBB (Fig. 4A and B; **P ≤ 0.01). Interestingly, numerous bright fluorescent spots were detected in the brains of SOD1^G93A^ mice, corresponding to areas of BBB impairment, while such spots were absent in sham mice. With a BBB score of 3.75, ALS04 is unlikely to cross an intact BBB (Fig. 1C), further supporting that these signals are due to BBB hyperpermeability in ALS mice. These findings demonstrate that ALS04 is a valuable imaging tool for detecting pathological changes in mitochondrial function in peripheral tissues and BBB disruption in the CNS of ALS mouse models.

## Discussion

ALS represents a formidable challenge in the field of neurodegenerative diseases, characterized by the progressive degeneration of both upper and lower motor neurons, ultimately leading to voluntary muscle atrophy and paralysis. The complexity of ALS progression, marked by genotypic diversity and phenotypic variability, presents hurdles in achieving accurate diagnoses and implementing effective therapeutic interventions. To address these challenges, we established for the first time a rationally designed NIR fluorophore for noninvasive monitoring of ALS disease progression. Our study particularly emphasizes the exploration of early-stage ALS biomarkers, with a particular focus on mitochondrial dysfunction and BBB breakdown as key indicators for disease monitoring (Fig. 1A).

Our results demonstrate that ALS04, a fluorophore that selectively localizes and accumulates in mitochondria, can effectively monitor mitochondrial function through NIR fluorescence imaging. We further show that ALS04 can detect mitochondrial dysfunction in an established ALS mouse model. This suggests that ALS04 is a valuable tool for noninvasive imaging of ALS biomarkers, providing insights into disease dynamics and potential therapeutic targets. By utilizing NIR fluorescence imaging, which offers deep tissue penetration and reduced nonspecific uptake, ALS04, designed based on the FDA-approved heptamethine indocyanine structure of ICG, emerges as a novel contrast agent for ALS monitoring. Given the limited monitoring methods currently available for ALS progression, our study opens a pathway for the development of a novel tool capable of monitoring ALS progression, enabling timely interventions to improve patient prognosis.

However, there are several limitations to the present study. First, our focus is primarily on validating ALS04 in animal models, specifically its biodistribution and mitochondrial accumulation. These findings may not directly translate to human ALS patients, and further validation in human samples or clinical trials is necessary. Second, ALS heterogeneity presents challenges in biomarker identification and disease monitoring. Biomarkers such as mitochondrial dysfunction and BBB breakdown may exhibit variability across ALS subtypes and disease stages, potentially affecting the reliability of ALS04 as a universal monitoring tool. Finally, while ALS04 shows promise as a diagnostic tool, its therapeutic efficacy in mitigating ALS progression has not been explored in this study. Further research is needed to determine whether ALS04 could also serve as a therapeutic agent in addition to its diagnostic potential.

## Conclusion

In summary, the mitochondria-targeted NIR fluorophore ALS04 offers a promising new tool for monitoring ALS biomarkers, enabling real-time fluorescence imaging in both peripheral tissues and the CNS in vivo. This innovative approach provides a means to investigate mitochondrial dysfunction in neurodegenerative diseases, offering valuable insights into pathophysiological changes. ALS04 holds potential for facilitating timely patient interventions and precise evaluations of disease states. The findings presented in this study pave the way for future research and clinical applications aimed at improving the diagnosis, monitoring, and therapeutic strategies for ALS and related neurodegenerative disorders.

## Ethical Approval

All animal procedures were performed in accordance with the Public Health Service Policy on Humane Care of Laboratory Animals and approved by the MGH IACUC (#2016N000136).

## Data Availability

The datasets and materials used and/or analyzed during the current study are available from the corresponding author upon reasonable request.
