# Supplementary material for: Image-Guided Monitoring of Mitochondria and Blood–Brain Barrier Dysfunction in Amyotrophic Lateral Sclerosis Mice
Source: Biomater Res. 2025 Mar 17;29:0162. doi: 10.34133/bmr.0162 (PMC11912748; doi:10.34133/bmr.0162)
Supplement: Supplementary 1 — Supplementary Methods Figs. S1 to S4 [file bmr.0162.f1.docx]

**Supporting information**

**Image-guided monitoring of mitochondria and blood-brain barrier dysfunction in amyotrophic lateral sclerosis (ALS) mice**

Do Won Hwang^1,2,*^, Jinhui Ser^1,3,*^, Konstantyn Zyabrev^4^, G. Kate Park^1^, Min Joo Jo^1^, Shinya Yokomizo^1^, Kai Bao^1^, Atsushi Yamashita^1^, Hoonsung Cho^3,**^, Maged Henary^1,**^, Satoshi Kashiwagi^1,**^, Hak Soo Choi^1,**^

^1^Gordon Center for Medical Imaging, Department of Radiology, Massachusetts General Hospital and Harvard Medical School, Boston, MA 02114, USA

^2^Research and Development Center, THERABEST Co., Ltd., 41, Seocho-daero 40-gil, Seocho-gu, Seoul 06656, South Korea

^3^Department of Materials Science and Engineering, Chonnam National University, Gwangju 61186, South Korea

^4^Department of Chemistry, Center of Diagnostics and Therapeutics, Georgia State University, Atlanta, GA 30303, United States

*These authors contributed equally to this work.

**Correspondence: skashiwagi@mgh.harvard.edu (S.K.); cho.hoonsung@jnu.ac.kr (H.C.); mhenary1@gsu.edu (M.H.); hchoi12@mgh.harvard.edu (H.S.C.)

The file includes:

**Supplementary Methods**

**Figure S1.** Chemical analyses of ALS04: NMR spectroscopy and HPLC chromatography.

**Figure S2.** Chemical analyses of ALS05: NMR spectroscopy and HPLC chromatography.

**Figure S3**. Cytotoxicity study of ALS04 and ALS05 in SH-SY5Y and NIH3T3 cells.

**Figure S4**. Cellular uptake study of ALS04 and ALS05 in SH-SY5Y cells.

**Supplementary Methods**

**Synthesis and characterization of ALS-targeted heptamethine cyanine fluorophores.**

All chemicals and solvents were purchased from Fisher Scientific (Pittsburgh, PA, USA), Sigma-Aldrich (Saint Louis, MO), Combi-Blocks (San Diego, CA), and Acros Organics. Both ^1^H- and ^13^C-NMR spectra were obtained using high-quality Kontes NMR tubes (Kimble Chase, Vineland, NJ) rated to 500 MHz and were recorded on a Bruker Avance (400 MHz) spectrometer using DMSO-*d*_6_ containing tetramethylsilane (TMS) as an internal calibration standard set to 0.0 ppm. NMR abbreviations used throughout the experimental section are as follows: s = singlet, d = doublet, t = triplet, q = quartet, p = pentet, m = multiplet, dd = doublet doublets, and bs = broad singlet. UV-Vis/NIR absorption spectra were recorded on a Varian Cary 50 spectrophotometer. Chemical purity was also confirmed using high-performance liquid chromatography (HPLC, Waters, Milford, MA) combined with photodiode array (PDA) and ESI-TOF mass.

*3-methyl-2-(7-(3-methylbenzothiazol-2(3H)-ylidene)-4,4’,5,6-tetrahydronaphthalen-2(3H)-ylid-enel)benzothiazolium tosylate* (**4; ALS04**): 0.26 g. Yield 41%, m.p. 232-234 °C. ^1^H-NMR (DMSO-*d*_6_ at 60 °C): 7.86 (d, J = 8.0 Hz, 2H), 7.58-7.46 (m, 6H), 7.29 (t, J = 8.0 Hz, 2H), 7.09 (d, J = 8.0 Hz, 2H), 6.40 (s, 2H), 6.27 (s, 2H), 3.76 (s, 6H), 2.88 (d, J = 16.0 Hz, 2H ), 2.61 (t, J = 16.0 Hz, 2H), 2.50 (DMSO+1H), 2.28 (s, 3H), 2.06 (d, J = 12.0 Hz, 2H), 1.44 (q, J = 12.0 Hz, 2H). ^13^C-NMR (DMSO-*d*_6_ at 60 °C): 160.04, 156.81, 156.76, 146.03, 140.38, 136.94, 127.63, 127.53, 125.23, 123.79, 122.11, 120.06, 112.37, 100.63, 34.40, 33.13, 29.90, 28.77, 20.35.

*6-methoxy-2-(7-(6-methoxy-3-methylbenzothiazol-2(3H)-ylidene)-4,4’,5,6-tetrahydro-naphthalen-2(3H)-ylidene)-3-methylbenzothiazolium tosylate* (**5; ALS05**): 0.116 g. Yield 17%, m.p. 170-171 °C. ^1^H-NMR (DMSO-*d*_6_): 7.48 (d, J = 8.0 Hz, 2H), 7.43-7.39 (m, 4H), 7.11 (d, J = 8.0 Hz, 2H), 6.98-6.95 (m, 2H), 6.24 (s, 2H), 6.17 (s, 2H), 3.75 (s, 6H), 3.68 (s, 6H), 2.81-2.77 (m, 2H), 2.50 (1H+DMSO+2H), 2.44-2.37 (m, 1H), 2.28 (s, 3H), 2.05-2.02 (m, 2H), 1.45-1.33 (m, 2H). ^13^C-NMR (DMSO-*d*_6_): 158.75, 156.09, 155.74, 155.54, 145.65, 137.31, 134.37, 127.81, 126.51, 125.29, 119.75, 114.86, 113.03, 106.42, 100.43, 55.56, 34.07, 33.24, 29.50, 28.84, 20.57.

**
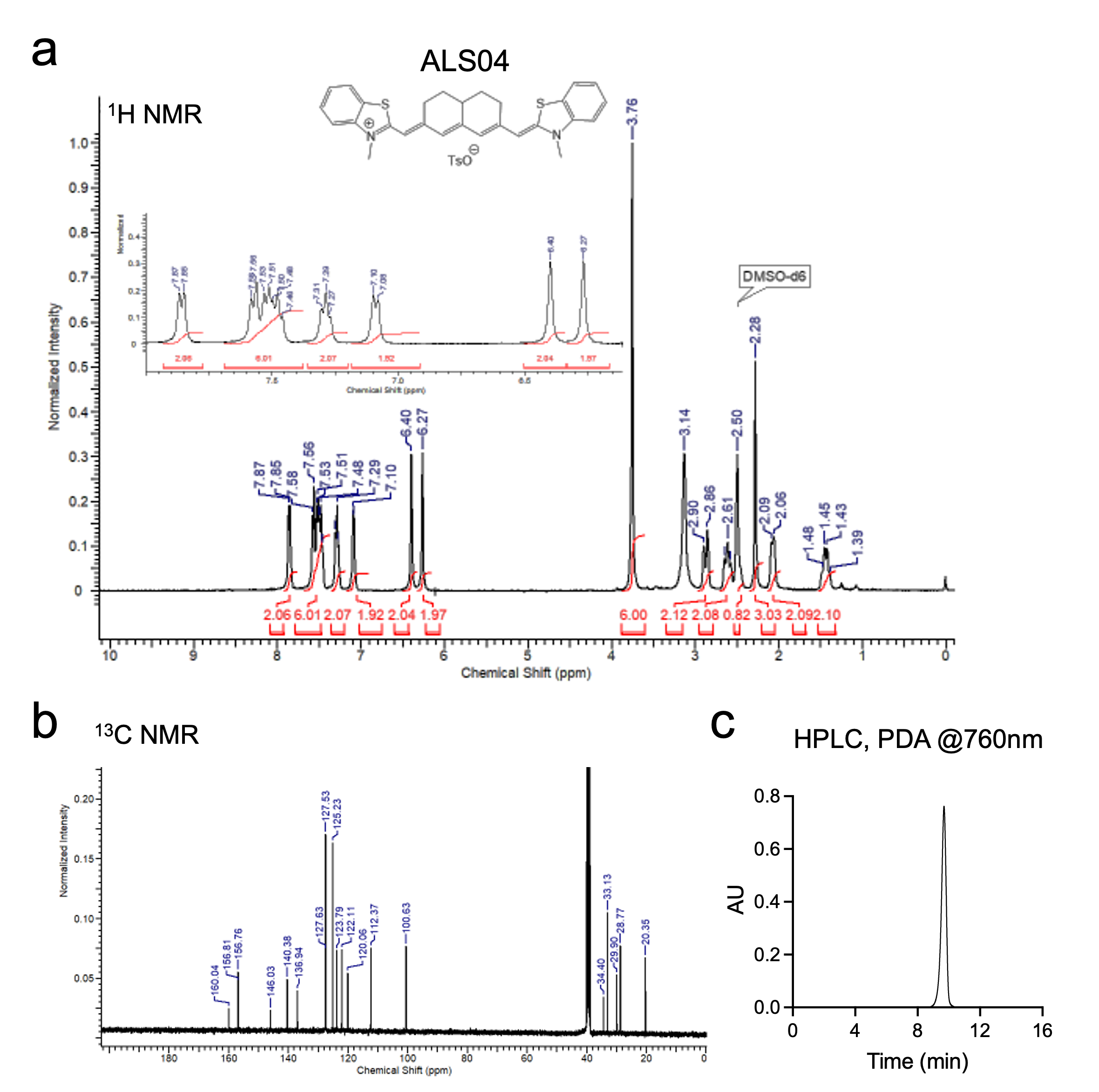
**

**Figure S1.** **Chemical analyses of** **ALS04**: (a) ^1^H-NMR spectroscopy, (b) ^13^C-NMR spectroscopy, and (c) HPLC chromatography with PDA @760 nm.


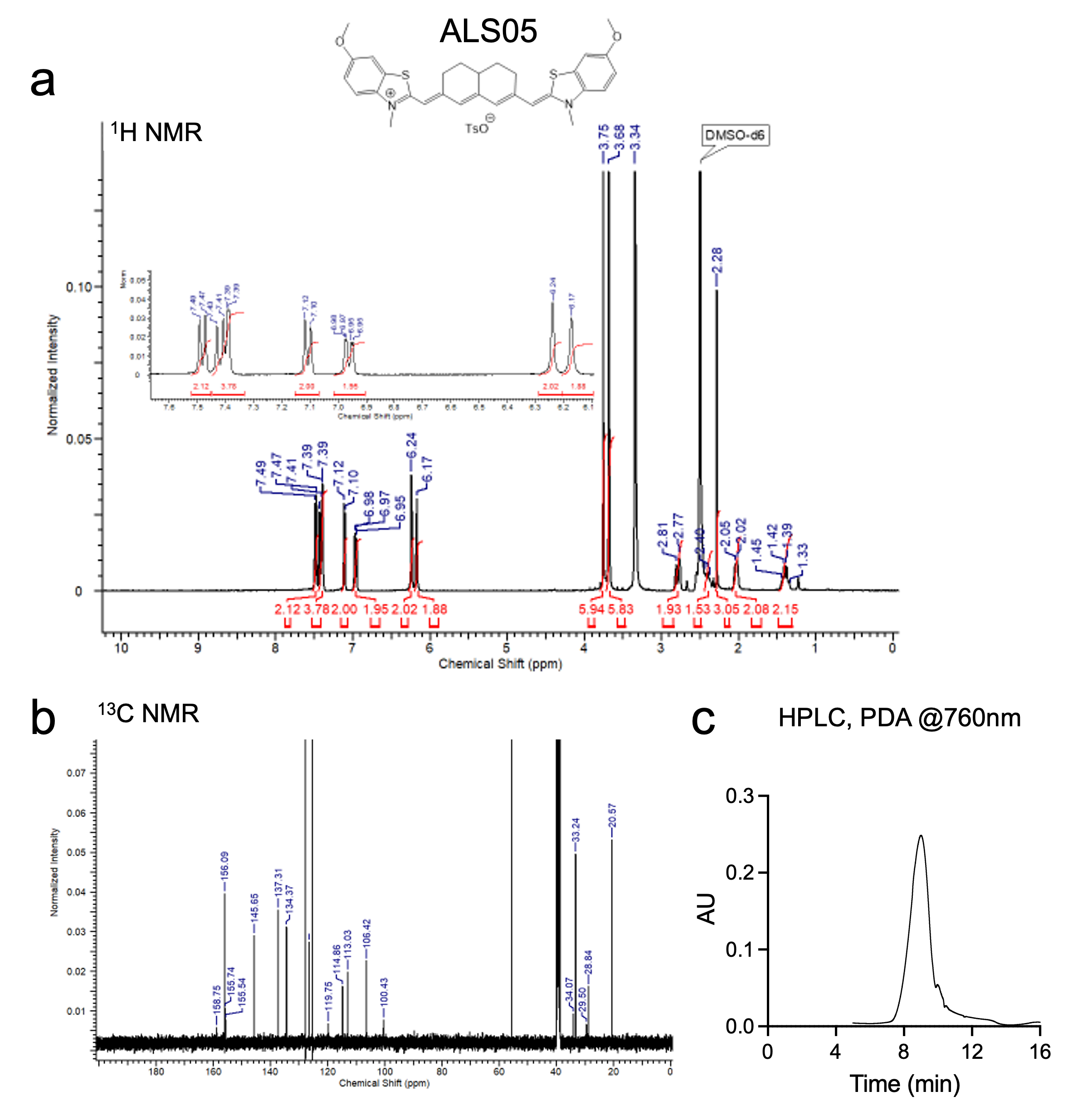


**Figure S2.** **Chemical analyses of** **ALS05**: (a) ^1^H-NMR spectroscopy, (b) ^13^C-NMR spectroscopy, and (c) HPLC chromatography with PDA @760 nm.


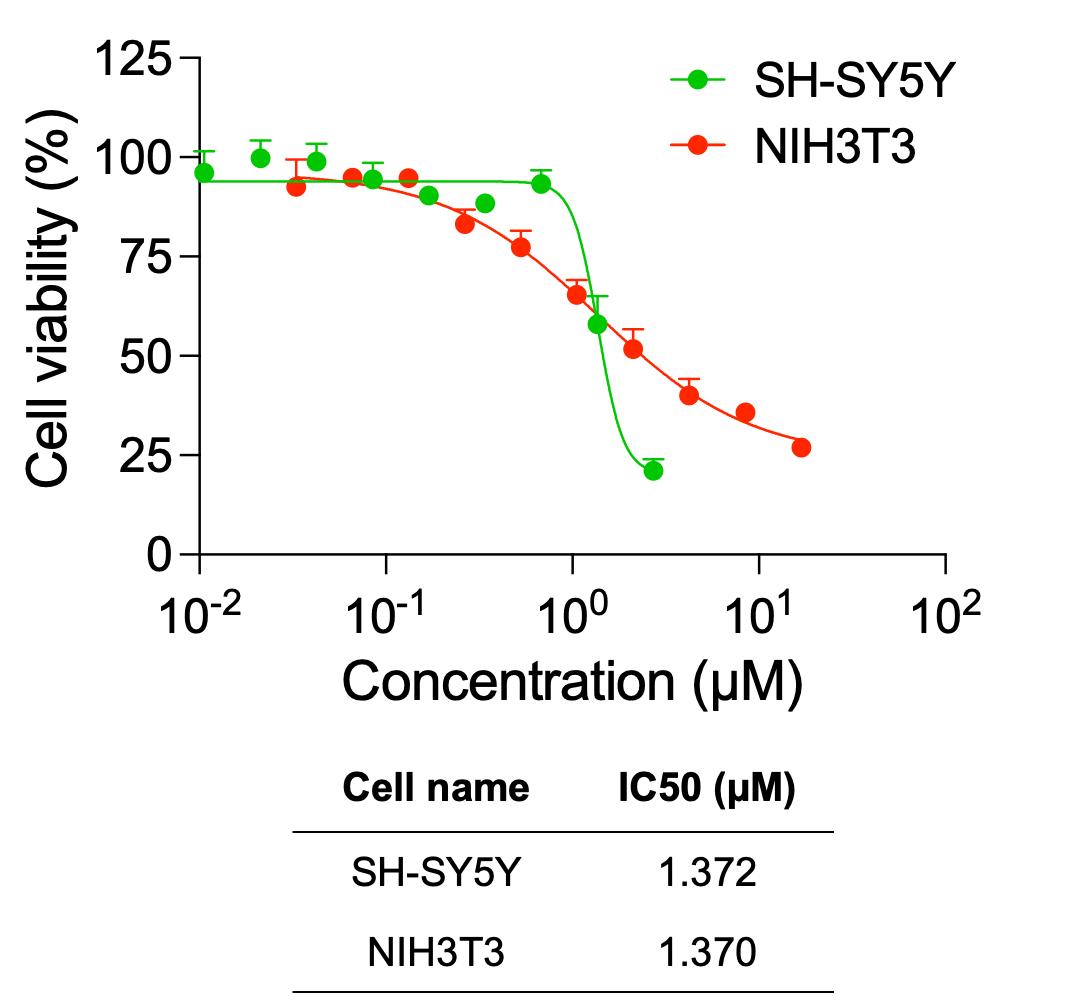


**Figure S3**. **Cytotoxicity study of ALS04 in SH-SY5Y and NIH3T3 cells.** Cells were treated with 0-20 μM of each fluorophore for 24 h (*n* = 5, mean ± SD), and cell viability was assessed using the Cell Counting Kit-8 (CCK-8).


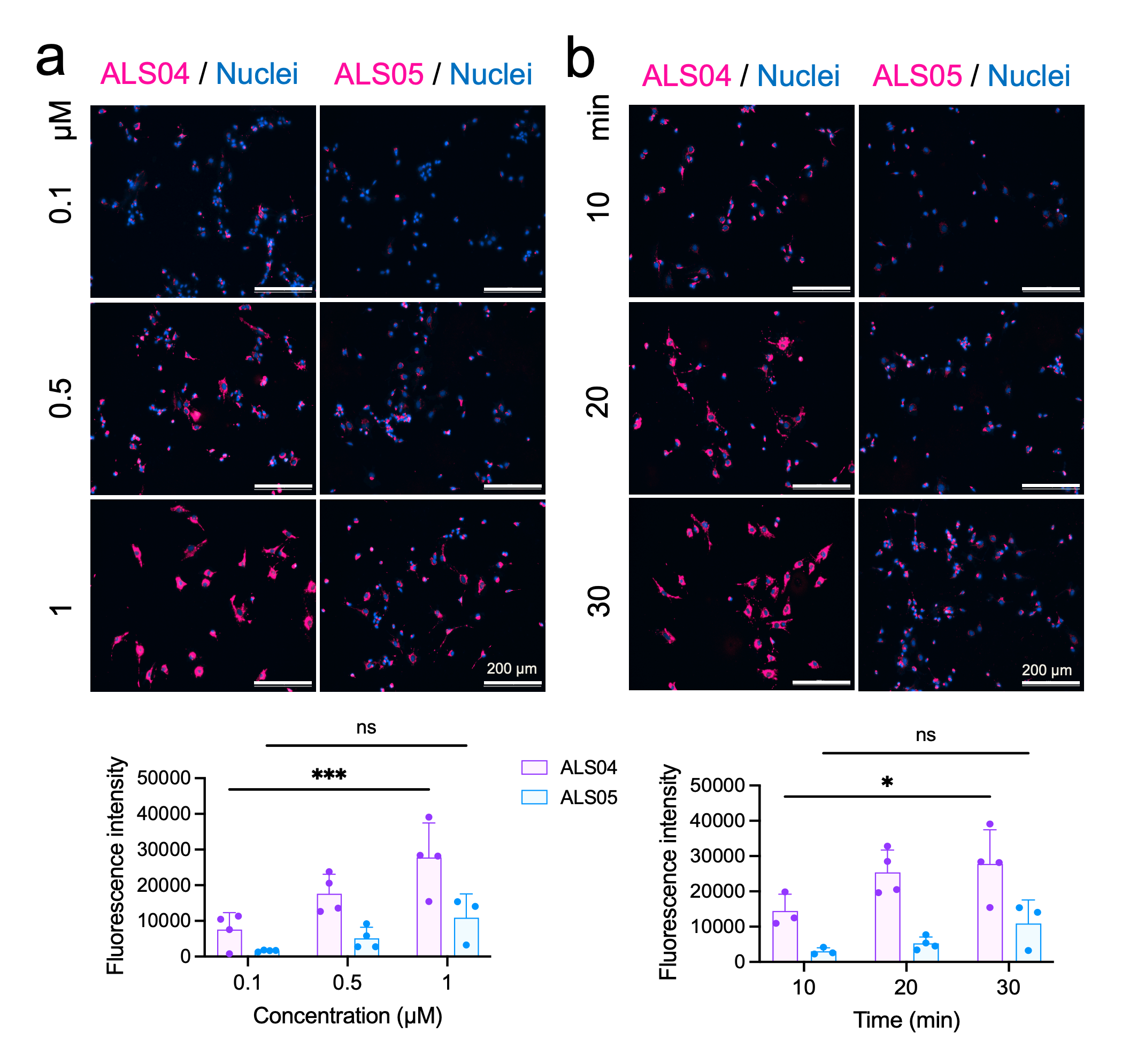


**Figure S4. Cellular uptake study of ALS04 and ALS05 in SH-SY5Y cells.** (a-b) SH-SY5Y cells were cultured and incubated for 10, 20, and 30 min in growth media containing 0.1-1 µM of ALS04 or ALS05, followed by imaging. Scale bar = 200 µm. The fluorescence intensity of cells in each group was quantified using the threshold analysis method in ImageJ software. Data were analyzed using two-way ANOVA, followed by Sidak’s multiple comparison test (*n* = 3-4, mean ± SD).
